# Analysis of Failure Mechanism of Silicone Gel Under High Voltage and High Temperature Aging Conditions

**DOI:** 10.3390/gels12080673

**Published:** 2026-07-27

**Authors:** Jiahui Zhang, Dongxin He

**Affiliations:** 1 School of Electrical Engineering, Shandong University, Jinan 250002, China; 15668463363@163.com; 2 Shandong Provincial Key Laboratory of UHV, Transmission Technology and Equipment, School of Electrical Engineering, Shandong University, Jinan 250002, China

**Keywords:** aging, electrical tree, electrical-thermal failure, molecular vibration, silicone gel

## Abstract

As a widely used encapsulant for power electronic devices, silicone gel is continuously exposed to high voltage and high temperature during service, which seriously impairs the reliability and service lifetime of power modules. This work investigates the electrothermal coupling failure mechanism of conventional silicone gel under high-voltage and high-temperature environments; the influences of different pulse electric field edge times and aging stages on various properties of the material are investigated, including electrical treeing characteristics, breakdown field strength, amplitude of charge-excited molecular vibration, leakage current, and cone penetration. It is revealed that the failure mechanism of silicone gel is attributed to the synergistic effect between dynamic charge damage induced by the pulse edge electric field and the degradation of the solid–liquid two-phase structure at high temperatures. This research provides theoretical support and experimental basis for material composition modification, structural optimization, and improving the encapsulation life of power electronic devices.

## 1. Introduction

Power semiconductor devices serve as the core components for electric energy conversion in modern industries, determining the energy conversion efficiency, power density, and reliability of power equipment. Silicone gel is an essential encapsulation and insulation material for power devices. As a composite insulation material, it offers excellent electrical insulation, ultra-high temperature resistance, elasticity, and viscosity after curing [1], along with favorable flame retardancy, chemical corrosion resistance and certain self-healing capacity [2]. However, it is frequently subjected to the combined electro-thermal effect of high voltage and high temperature during service, which severely jeopardizes the reliability and service life of silicone gel encapsulation. Therefore, in-depth exploration of the electrical and thermal failure mechanisms of traditional silicone gel is key to proposing relevant material modification schemes.

Domestic and foreign scholars have carried out extensive research on the electrical and thermal performance evolution of silicone gel and polymer insulation materials. In terms of insulation performance under pulse electric field, researchers have explored the influence of pulse duration, frequency and polarity on the initiation and growth of electrical trees and found that same-polarity DC accelerates tree discharge, the electrical tree initiation voltage is lower under positive pulses, and the increase in pulse frequency increases the electrical tree density [3,4,5,6]. Nakamura et al. further found that filamentous electrical trees are easily formed in silicone gel under positive pulses, spindle-shaped under negative pulses, and the length of electrical trees increases with the increase in frequency [7]. Despite these advances, the influence of electrothermal coupling on electrical tree evolution remains insufficiently understood. Additionally, few studies have been conducted on the key parameter of nanosecond-scale pulse edge time, and the regulatory mechanism by which the voltage change rate induced by edge time governs charge dynamic behavior and electrical tree development remains unelucidated.

In view of the above research limitations, this paper systematically investigates the electrothermal coupling failure mechanism of conventional silicone gel under high-voltage and high-temperature service conditions. A thermal aging temperature of 240 °C was adopted to characterize the staged evolution of the solid–liquid biphasic structure of conventional silicone gel under prolonged high-temperature exposure. The experimental results are analyzed from two dimensions, namely the microscopic dynamic behavior of space charges and the macroscopic structural degradation of materials. The mutually promoting interaction mechanism between charge dynamic damage induced by pulsed electric fields and structural deterioration caused by high temperature is elaborated in detail. The findings of this work lay a theoretical foundation for developing anti-aging and pulse-breakdown-resistant silicone encapsulant gels and also provide engineering references for reliability optimization of internal packaging structures of high-voltage IGBTs and power modules.

## 2. Results and Discussion

### 2.1. Electrical and Thermal Performance Test and Analysis

#### 2.1.1. Influence of Different Edge Times on the Initiation and Development of Electrical Tree in Silicone Gel

**Figure 1:**
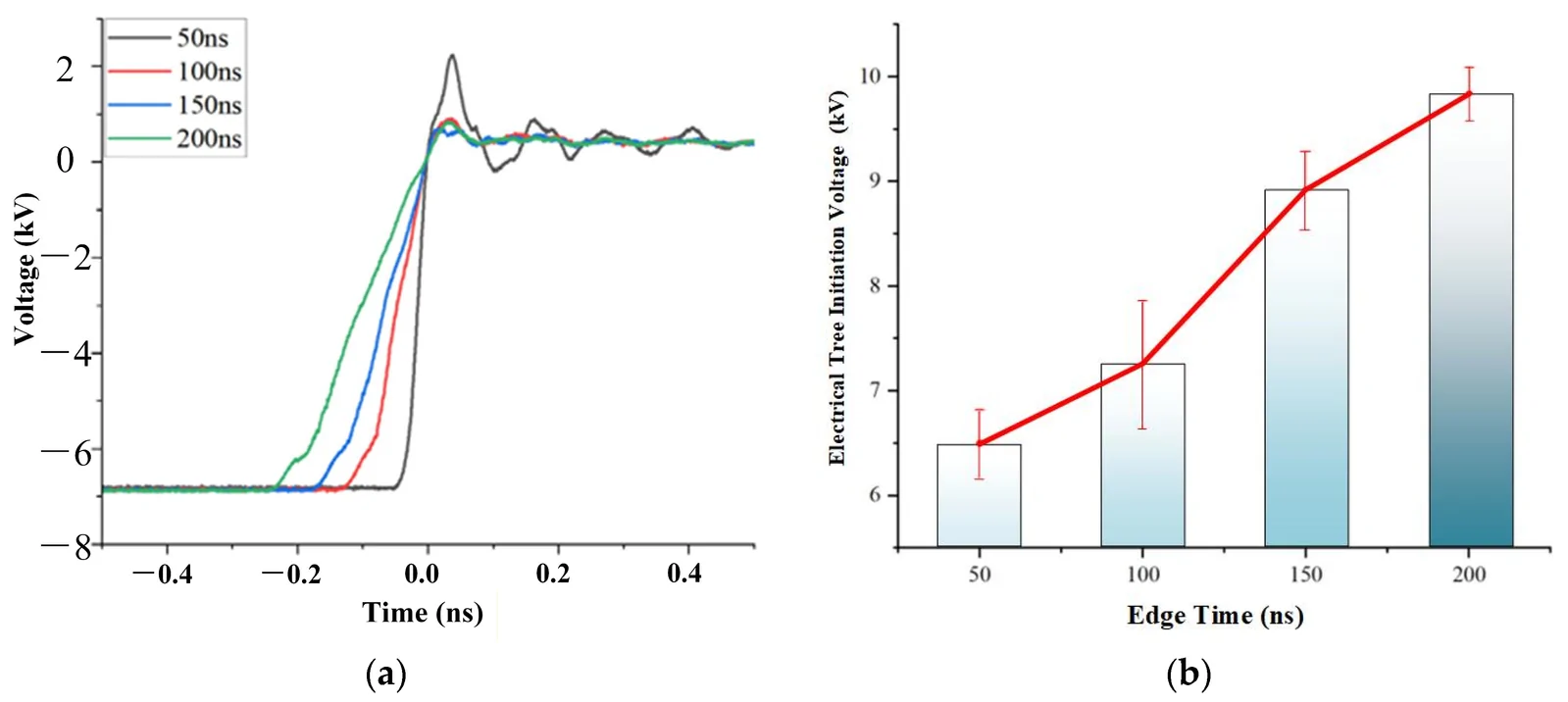
(**a**) Square-wave pulse voltage waveforms with different edge times. (**b**) Initial voltage of electrical tree with different edge times.

To explore the influence of the electric field change rate at the edge on the initiation and development of electrical tree, experiments with different edge times were designed to record the initial voltage of electrical tree and observe the development and change in its micro-morphology. In this study, the tree inception voltage (TIV) is defined as the applied voltage at which filamentous electrical trees with a length of ≥5 μm first appear at the needle tip, observed by an optical microscope, when the silicone gel sample is subjected to a nanosecond-level pulse electric field. During the measurement, the pulse voltage is gradually increased at a rate of 100 V/step, maintained for 10 min each time. Immediately after the inception of the electrical tree, the voltage is cut off and the inception voltage is recorded. Each group of experiments is repeated five times, and the average value is taken. The pulse electric field edge times were set to 50 ns, 100 ns, 150 ns, and 200 ns. The experimental waveforms and results are shown in Figure 1.

The experimental results above demonstrate that tree inception voltage increases with extended pulse edge time, though the rising trend slows gradually rather than following an exponential growth curve. In addition, a shorter pulse edge time corresponds to a higher voltage change rate. As a result, the interference of parasitic circuit parameters (stray inductance L and stray capacitance C, both inherent to the experimental circuit) on voltage waveforms becomes more pronounced. The mismatch between circuit parasitic effects and the charge relaxation process inside dielectrics further induces severe voltage overshoot and local electric field distortion [8,9]. An obvious overshoot phenomenon occurs at an edge time of 50 ns, while at a longer edge time, the charge relaxation is sufficient and the electric field distortion is weakened, resulting in mild voltage overshoot on waveforms. This difference in overshoot further leads to the experimental result that the electrical tree inception voltage increases with the extension of the edge time [10,11].

**Figure 2:**
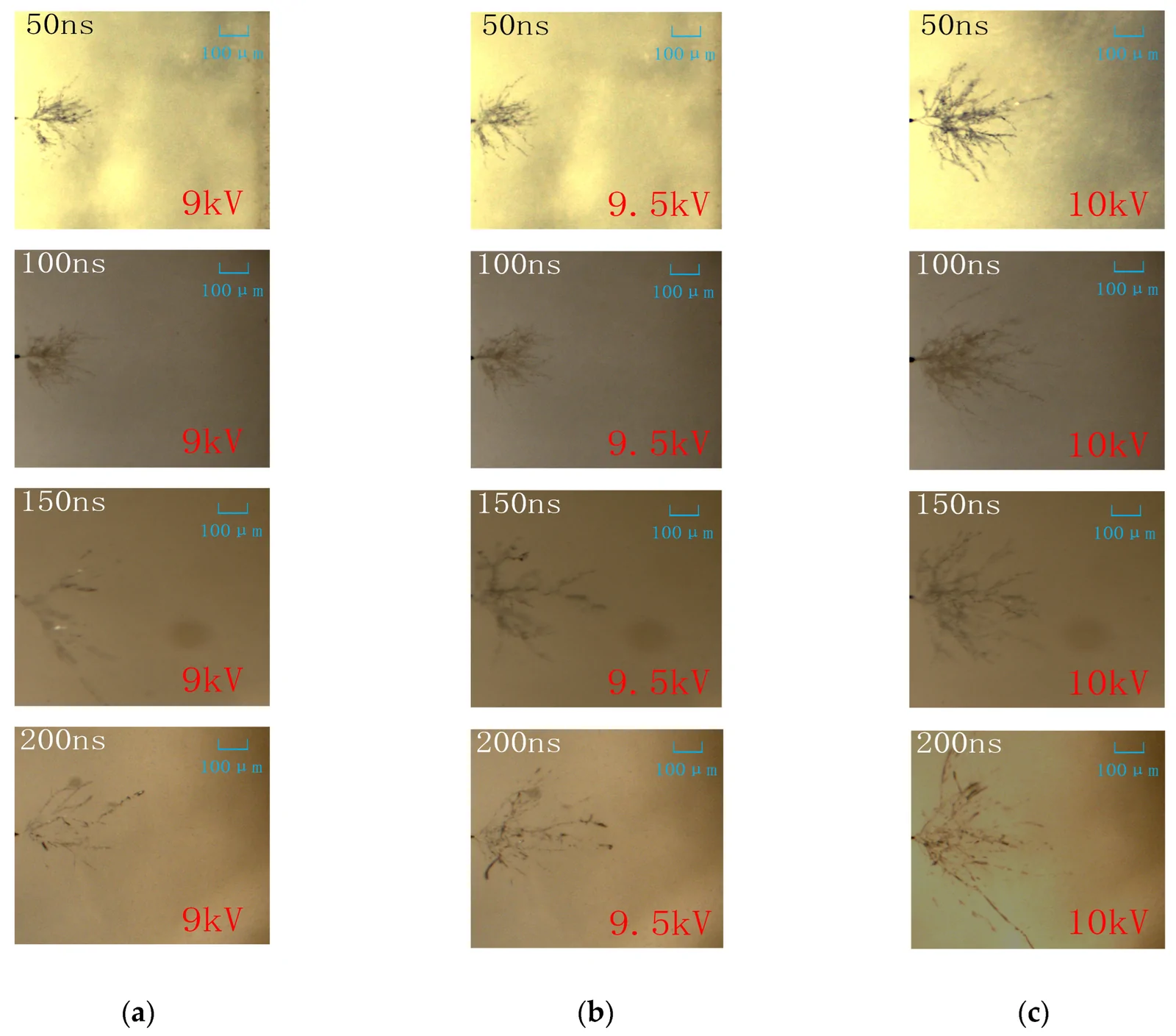
Changes in the micro-morphology of electrical tree under different edge times. (**a**) Electrical tree morphology under 9 kV with different edge times; (**b**) electrical tree morphology under 9.5 kV with different edge times; (**c**) electrical tree morphology under 10 kV with different edge times.

Comprehensively considering factors such as the initial voltage under different conditions, the binarized images of the electrical tree at voltage amplitudes of 9 kV, 9.5 kV, and 10 kV were selected for analysis. The changes in the micro-morphology of the electrical tree under pulse electric field edge times of 50 ns, 100 ns, 150 ns, and 200 ns are shown in Figure 2.

The length of the electrical tree is defined as the distance along the electric field direction from the needle electrode to the farthest branch tip, and the width is defined as the distance between the two farthest branch tips perpendicular to the electric field direction, for comparative analysis. By comparing the morphology of electrical trees under the same voltage level and pressurization time with different edge times, it is found that with the increase in edge time, the branches become smaller and thinner, the tree inception voltage is higher, and the morphology of electrical trees is sparser. Although the shape is more divergent, the growth rate is slower, indicating that the increase in edge time has an inhibitory effect on the growth of electrical trees. Since the exact law cannot be obtained only by comparing the length and width of electrical trees. Image processing and quantitative calculation of electrical tree morphology are performed as described below.

**Figure 3:**
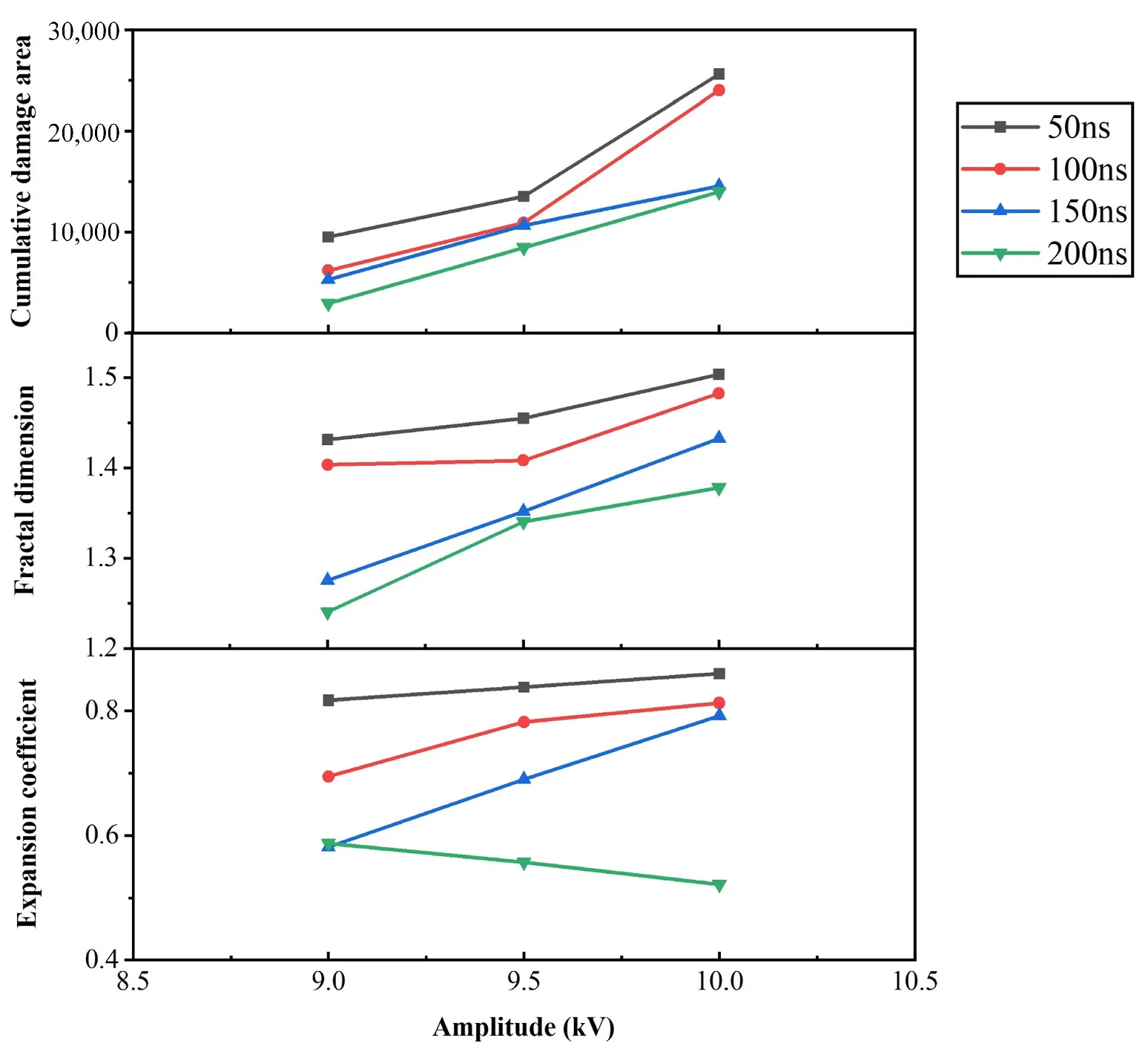
Fractal dimension, expansion coefficient, cumulative damage area of electrical tree under different edge times.

Figure 3 illustrates that under the same voltage, the longer the pulse edge time, the lower the fractal dimension and expansion coefficient of the electrical tree. It is speculated that, on the one hand, an electrical tree with longer edge times initiates only at higher voltages, resulting in insufficiently rapid growth of electrical tree length. On the other hand, when the pulse edge time is prolonged, the electron movement speed decreases, leading to even smaller growth in electrical tree length. It can also be observed from the figure that under the same voltage level, the cumulative damage area of the electrical tree is smaller when the edge time is larger, but it still shows an upward trend with the increase in voltage level. It is speculated that the shorter the pulse edge time, the greater the voltage change rate. On the one hand, a large number of charges are injected into the dielectric within an extremely short time, leaving insufficient time for complete charge relaxation. These charges migrate under the excitation of the rising and falling edges of the pulse voltage, triggering molecular collisions that directly induce the scission of molecular chains and form initial electrical tree channels [12]. On the other hand, the rapid voltage change induces a local temperature rise, softens polymer chains, reduces the material’s breakdown field strength, and further accelerates the growth of electrical trees, ultimately leading to aggravated electrical tree degradation.

When the edge time is short, electrical trees grow faster and denser. It is speculated that the increase in voltage variation rate intensifies electric field distortion, generating high-energy charged particles during discharge. Under the action of the applied electric field, these particles bombard molecular chains, resulting in bond breakage and interconnection of microscopic defects, thereby rapidly forming continuous growth channels for electrical trees inside the silicone gel [13]. In contrast, at longer edge times, the effects of all these processes are significantly weakened, exerting a certain inhibitory effect on the growth of electrical trees.

#### 2.1.2. Analysis of Breakdown Field Strength of Silicone Gel at Different Aging Stages

As encapsulation insulation for power electronic devices, conventional silicone gel is frequently subjected to high-temperature operating conditions, which can lead to significant changes in its physical, chemical, and electrical properties [14]. During thermal aging, the material undergoes a series of processes such as weight loss, reduction in relative molecular mass, melting, and changes in crystallinity and crosslinking degree under the influence of heat and other factors, resulting in performance degradation [15].

Thermogravimetric analysis (TGA) results indicate that conventional silicone gel exhibits negligible mass loss below 200 °C, accompanied by extremely subtle variations in its solid–liquid two-phase structure. Under short-duration thermal aging, obvious evolutions of characteristic properties including electrical tree morphology, breakdown field strength and cone penetration hardness cannot be captured, which makes it difficult to reveal the intrinsic aging and failure mechanisms of the material.

Within the temperature range of 230–250 °C, free cyclic siloxanes and low-molecular-weight silicone oil volatilize massively. This temperature interval precisely reproduces the real degradation process of encapsulation insulation under practical high-temperature operation, namely the imbalance of the solid–liquid two-phase structure and subsequent insulation deterioration.

By contrast, thermal decomposition of silicone gel occurs at approximately 300 °C, triggering severe irreversible damage such as material carbonization and complete molecular pyrolysis. Such extreme degradation phenomena deviate greatly from actual service failure modes of power modules; hence, these temperatures are unsuitable for experimental aging tests [16,17].

**Figure 4:**
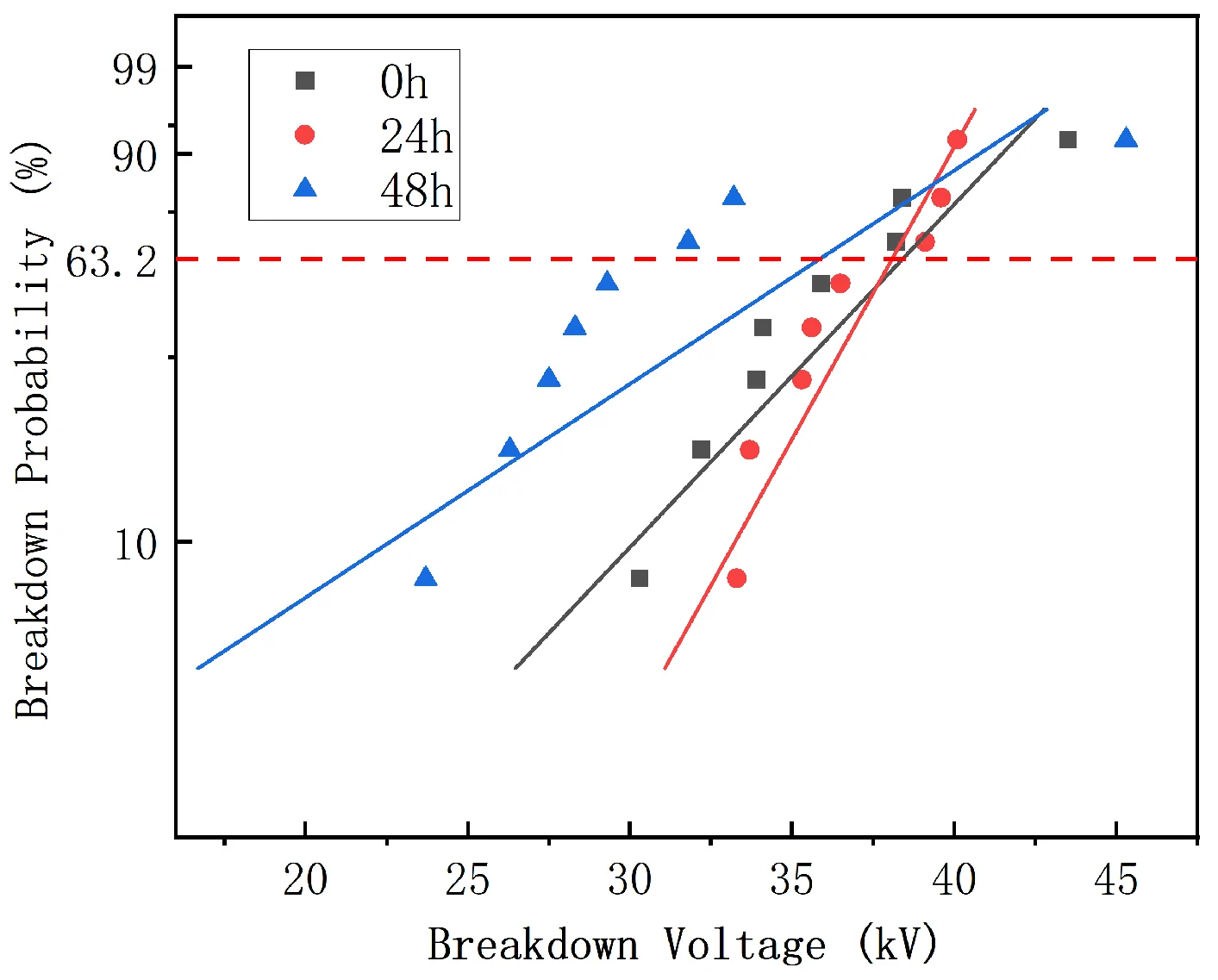
Weibull distribution of breakdown voltage under different aging times.

Accordingly, the aging temperature is set to 240 °C, with aging durations of 24 h and 48 h, respectively. All experimental results simulate the actual packaging insulation failure. The breakdown data were processed using the Weibull distribution, and Weibull plots of the breakdown voltage of silicone gel samples under different aging times were obtained. The breakdown voltage corresponding to a cumulative breakdown probability of 63.2% was taken as the characteristic breakdown voltage of the silicone gel samples, as shown in Figure 4.

All experimental results are simulations of actual encapsulation insulation failure results. As shown in Table 1, the characteristic breakdown field strength of unaged samples at 240 °C is 38.5 kV/mm, showing excellent insulation performance. After aging at 240 °C for 24 h, the breakdown field strength of the silicone gel decreases to 38.1 kV/mm, and the breakdown resistance exhibits no significant change. After aging at 240 °C for 48 h, the breakdown field strength decreases to 35.8 kV/mm. This indicates that in the initial state, the molecular chains of the silicone gel are highly ordered, so that under the action of the electric field, the polarization response inside the material is rapid and uniform, resulting in a high breakdown strength [18]. After short-term thermal aging, the internal microstructure of silicone gel becomes densified to a certain extent. Apart from crosslinking reactions, oxidation reactions simultaneously take place within the material system and generate abundant siloxane bonds, which increase the overall crosslink density and form a denser network structure. Consequently, the breakdown field strength barely changes at this stage. As aging proceeds further, massive loss of internal silicone oil occurs. High-temperature exposure induces bond scission and degradation of the crosslinking network, leading to the generation of numerous microcavities and microcracks inside the matrix. The proliferation of such void defects ultimately gives rise to severe insulation performance degradation [19].

In summary, on the one hand, the existence of bubbles leads to a decrease in local dielectric strength and an uneven electric field, accelerating partial discharge [20]. On the other hand, the emergence of internal micro-defects in the silicone gel and the significant degradation of its self-healing property jointly contribute to the variation in breakdown field strength after aging [21].

#### 2.1.3. Analysis of Charge-Excited Molecular Vibration Amplitude of Silicone Gel at Different Aging Stages

**Figure 5:**
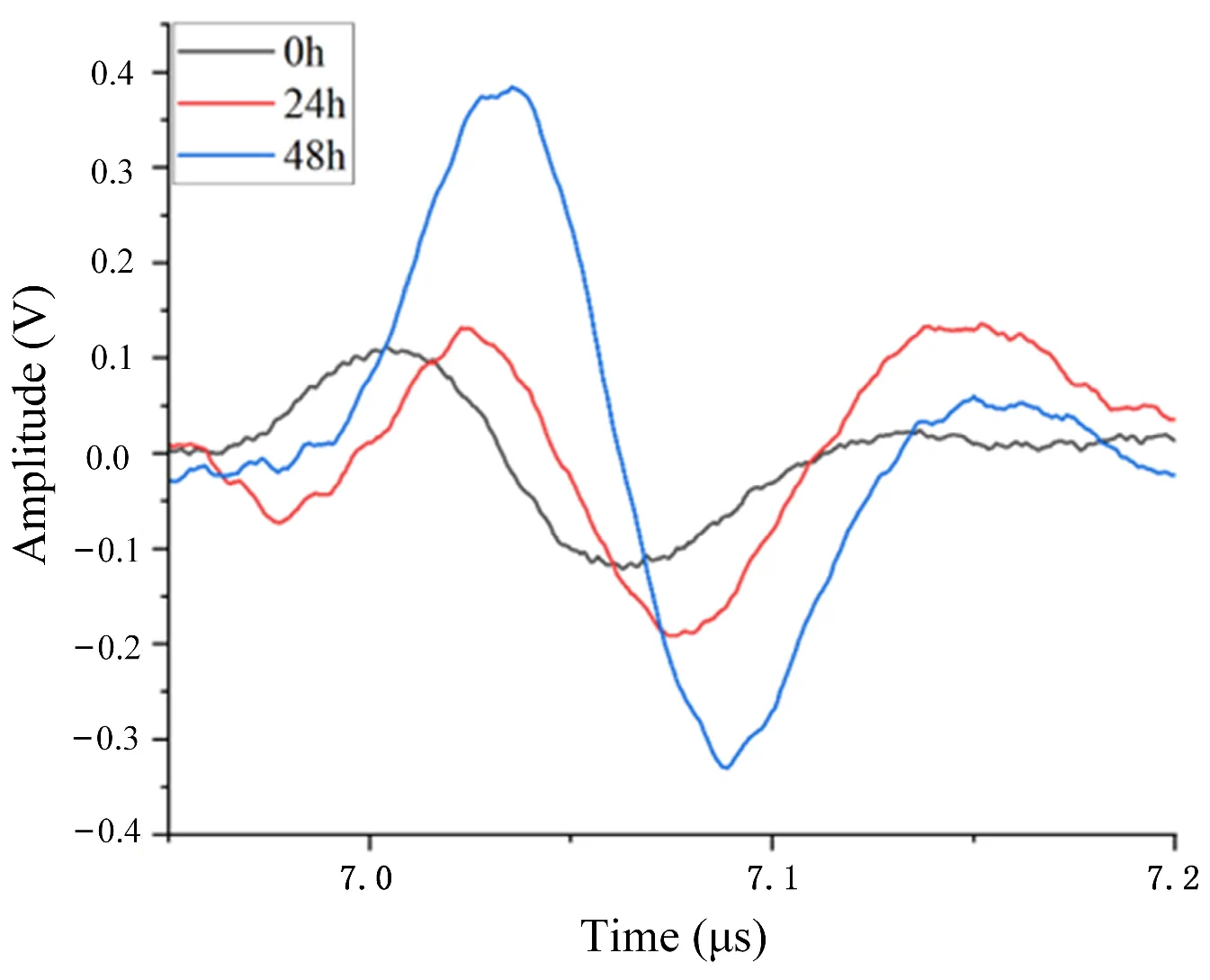
Charge-excited molecular vibration amplitude of silicone gel at different aging stages.

This section analyzes the charge-excited molecular vibration amplitude of silicone gel at different aging stages. The experimental results are shown in Figure 5. The charge-excited molecular vibration amplitude of samples aged at 240 °C for 24 h rises by a small margin. The underlying mechanisms are analyzed as follows. On the one hand, the crosslinking degree of silicone gel rises and the number of crosslinking nodes on molecular chains increases. The molecular vibration energy induced by charge impact is dissipated through the crosslinking network, thereby suppressing the vibration amplitude. On the other hand, prolonged aging introduces abundant microdefects inside the material, which promotes the growth of molecular vibration amplitude. These two competing effects counterbalance each other during short-term aging, resulting in an insignificant overall variation in vibration amplitude. When the aging condition is extended to 240 °C for 48 h, the charge-excited molecular vibration amplitude increases drastically, demonstrating that the amplitude enhancement effect originating from accumulated microdefects becomes the dominant factor.

The unique solid–liquid two-phase structure of silicone gel leads to two opposite trends after aging. On one hand, liquid-phase molecules participate in the post-crosslinking reaction and are gradually incorporated into the solid network, resulting in a decreased proportion of liquid components and an increased crosslinking density [21], which weakens the amplitude of molecular vibration excited by free electrons. On the other hand, aging gives rise to an increase in low-molecular impurities and microdefects within the material, which raises the quantity of mobile charge carriers inside the silicone gel and consequently amplifies the molecular vibration amplitude [22,23].

Two opposite effects coexist during thermal aging of silicone gel. The first is the crosslinking reinforcement effect. Short-term thermal aging triggers post-crosslinking of molecular chains to form a denser network, which restricts molecular motion and dissipates vibration energy, thus reducing the vibration amplitude. The second is the defect proliferation effect. High-temperature aging increases the density of both shallow and deep traps within silicone gel. These defect sites capture homopolar space charges and induce severe distortion of the local electric field. Meanwhile, elevated temperature enhances the intrinsic activity of molecular chains. Combined with the electrostatic force exerted by charges under the applied pulsed electric field, the stretching, twisting and vibration of molecular chains are markedly intensified.

After 24 h of short-term aging, the crosslink density is improved, and the binding constraint of the molecular network is strengthened, limiting the free movement space of molecular chains. As a result, the vibration amplitude only rises slightly. In contrast, 48 h of prolonged aging generates massive internal defects such as microvoids and microcracks, which trap abundant space charges. Local charge accumulation greatly amplifies the excitation effect. In addition, partial scission of molecular chains weakens the constraint from the crosslink network, leading to a remarkable increase in molecular vibration amplitude [24].

Meanwhile, the denser cross-linking network after aging restricts the motion of molecular chains, resulting in a slower charge relaxation rate and thus a phase lag in the vibration response [18]. As aging time increases, the occurrence time of the peak shifts slightly later.

#### 2.1.4. Analysis of Leakage Current of Silicone Gel at Different Aging Stages

**Figure 6:**
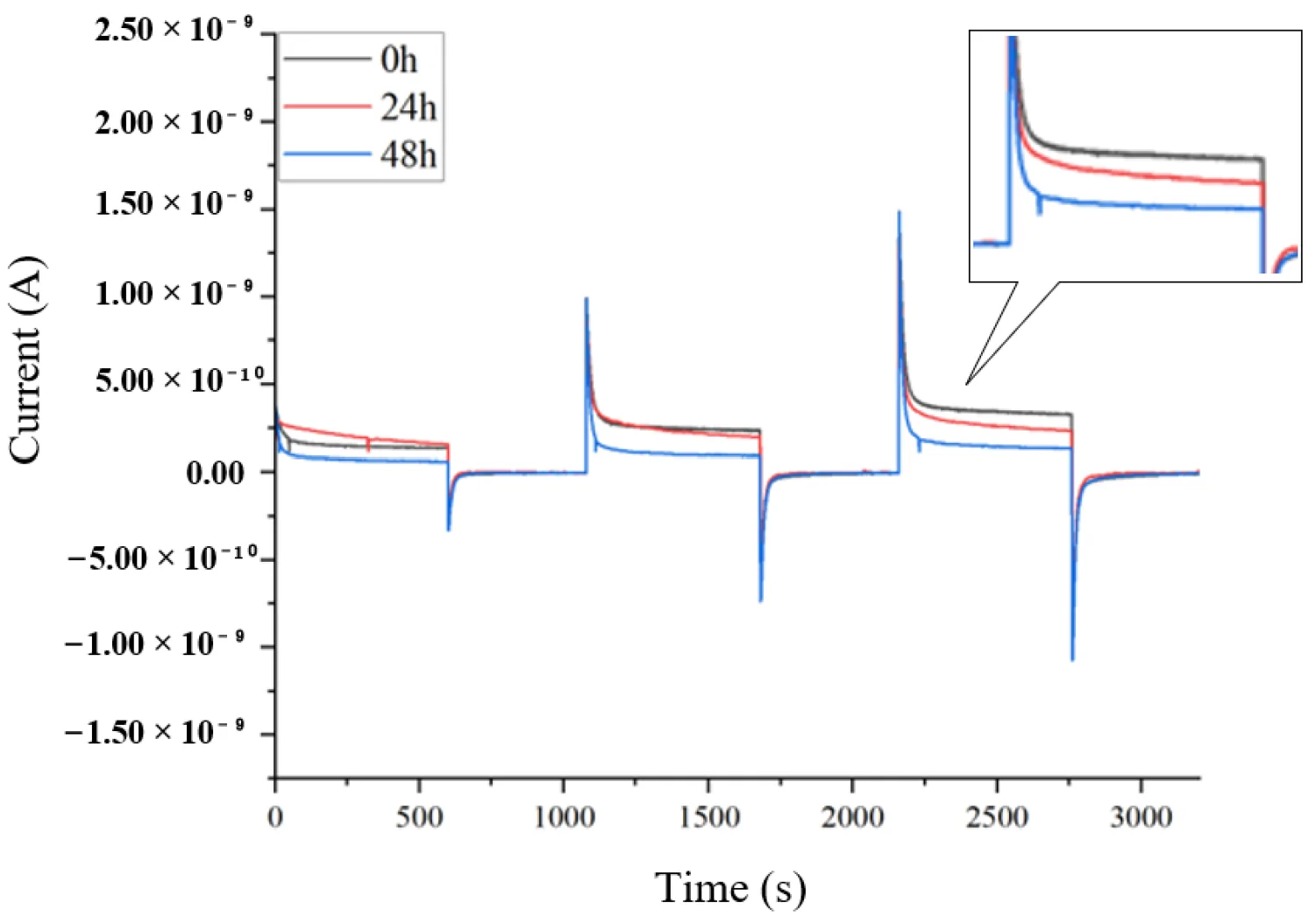
Leakage current of silicone gel at different aging stages.

Leakage current tests on silicone gel samples with different aging durations further verify the above experimental phenomena. The experimental results are shown in Figure 6.

The leakage current of unaged specimens under test temperature stays at an overall low level, which indicates that the original silicone gel contains few internal defects and conductive paths and closely adheres to the device wall. In this state, the material exhibits favorable insulation and encapsulation performance. After high-temperature thermal aging, the leakage current of samples rises significantly. This phenomenon verifies the foregoing conclusion that silicone oil volatilizes and the material becomes rigid, accompanied by interfacial gaps between the gel and device substrate. Internal defects accumulate continuously inside silicone gel, gradually developing into microcracks and voids. Meanwhile, part of the liquid-phase components volatilize and escape under persistent high temperature, while the remaining fraction participates in thermal oxidative crosslinking reactions and is incorporated into the solid crosslinked network. The drastic reduction in free low-molecular liquid silicone oil breaks the inherent solid–liquid two-phase equilibrium of silicone gel, greatly weakening its viscoelasticity and self-healing capability and eventually accelerating insulation failure under electrothermal synergy. Although the crosslinking degree and hardness increase synchronously, conductive leakage paths emerge both on the material surface and within the bulk [19]. Furthermore, Joule heat generated by leakage current passing through the dielectric further damages the molecular structure and reduces material resistivity, which in turn causes continuous growth of leakage current. A positive feedback degradation effect of electric–thermal coupling is therefore formed, drastically expediting the deterioration of insulation properties [25,26].

#### 2.1.5. Analysis of Electrical Tree Initiation Voltage and Micro-Morphology of Silicone Gel at Different Aging Stages

This section discusses the electrical tree initiation voltage and the development and change in its micro-morphology of silicone gel at different aging stages. The experimental results are shown in Table 2. The electrical tree initiation voltage of the sample aged at 240 °C for 0 h is 6.5 kV. After aging at 240 °C for 24 h, the electrical tree initiation voltage increases to 8.25 kV. This is attributed to the oxidation of organic side groups within the silicone gel after aging, which increases the crosslinking density and thus requires higher electrical stress to induce molecular chain scission [15].

With the further extension of aging time to 48 h, the electrical tree initiation voltage decreases to 7.8 kV. This is because although the crosslinking density of the silicone gel further increases at this time, aging also leads to the generation of internal micro-defects and small molecular impurities in the silicone gel, making it easier for partial discharge to occur inside. These two factors play opposite roles. Experimental results reveal that within 48 h aging duration, the improved insulating strength originating from an elevated molecular crosslinking degree dominates. However, it can be predicted that when the aging time is further extended and the liquid components in the silicone gel further disappear, the increase in crosslinking degree will gradually slow down, and the increase in internal micro-defects will dominate, leading to a significant decrease in the electrical tree initiation voltage of the silicone gel.

In summary, even though the electrical tree initiation voltage of silicone gel increases after a short period of aging, the intrinsic properties of the material do not undergo significant loss. Nevertheless, issues such as bubble accumulation, excessive leakage current, and increased surface discharge still lead to the premature failure of the silicone gel.

**Figure 7:**
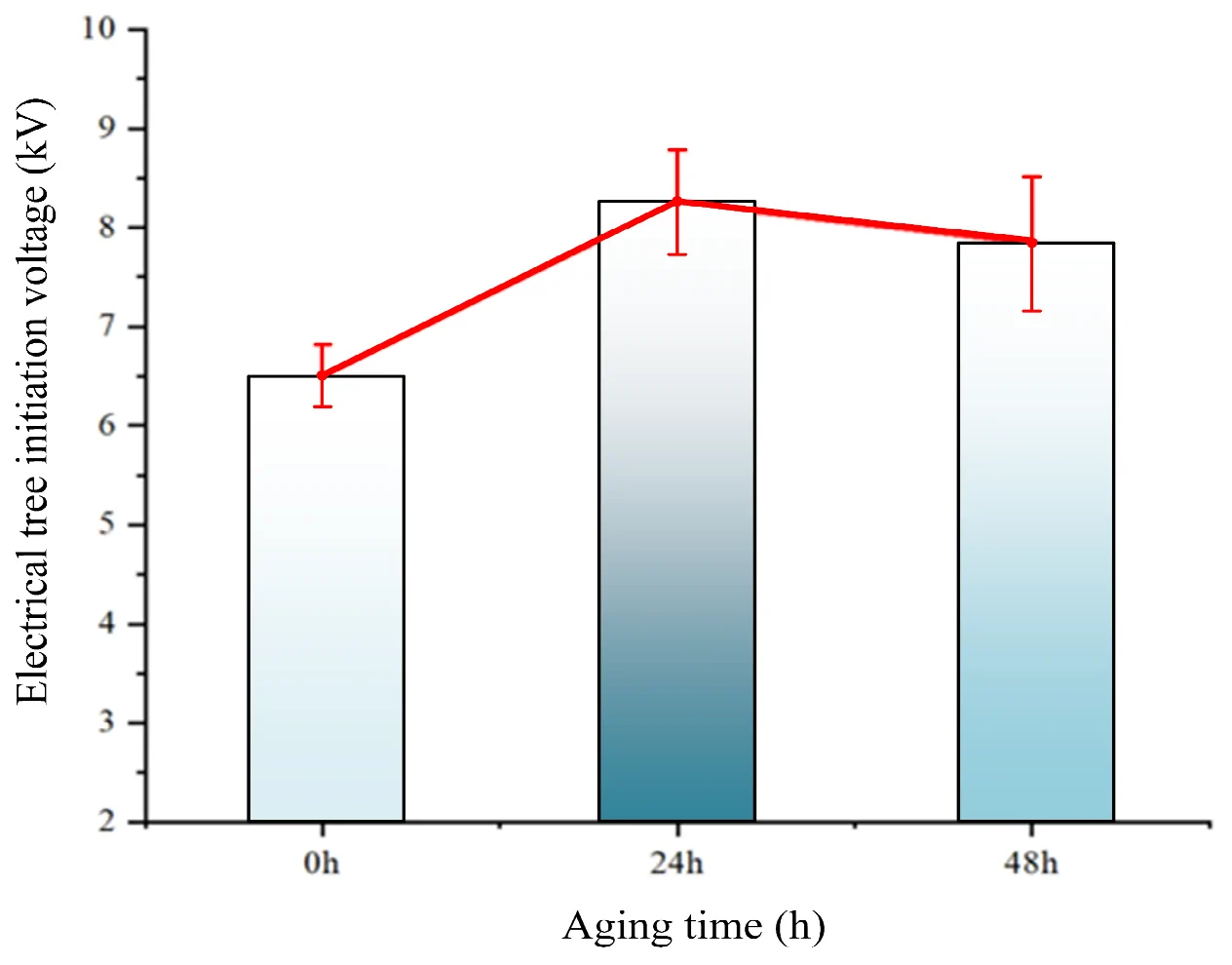
Electrical tree initiation voltage of silicone gel at different aging stages.

In addition, comprehensively considering factors such as the initiation voltage, the electrical tree images at different aging times under voltage amplitudes of 8 kV, 8.5 kV, 9 kV, 9.5 kV, and 10 kV were selected for analysis; the results are shown in Figure 7.

**Figure 8:**
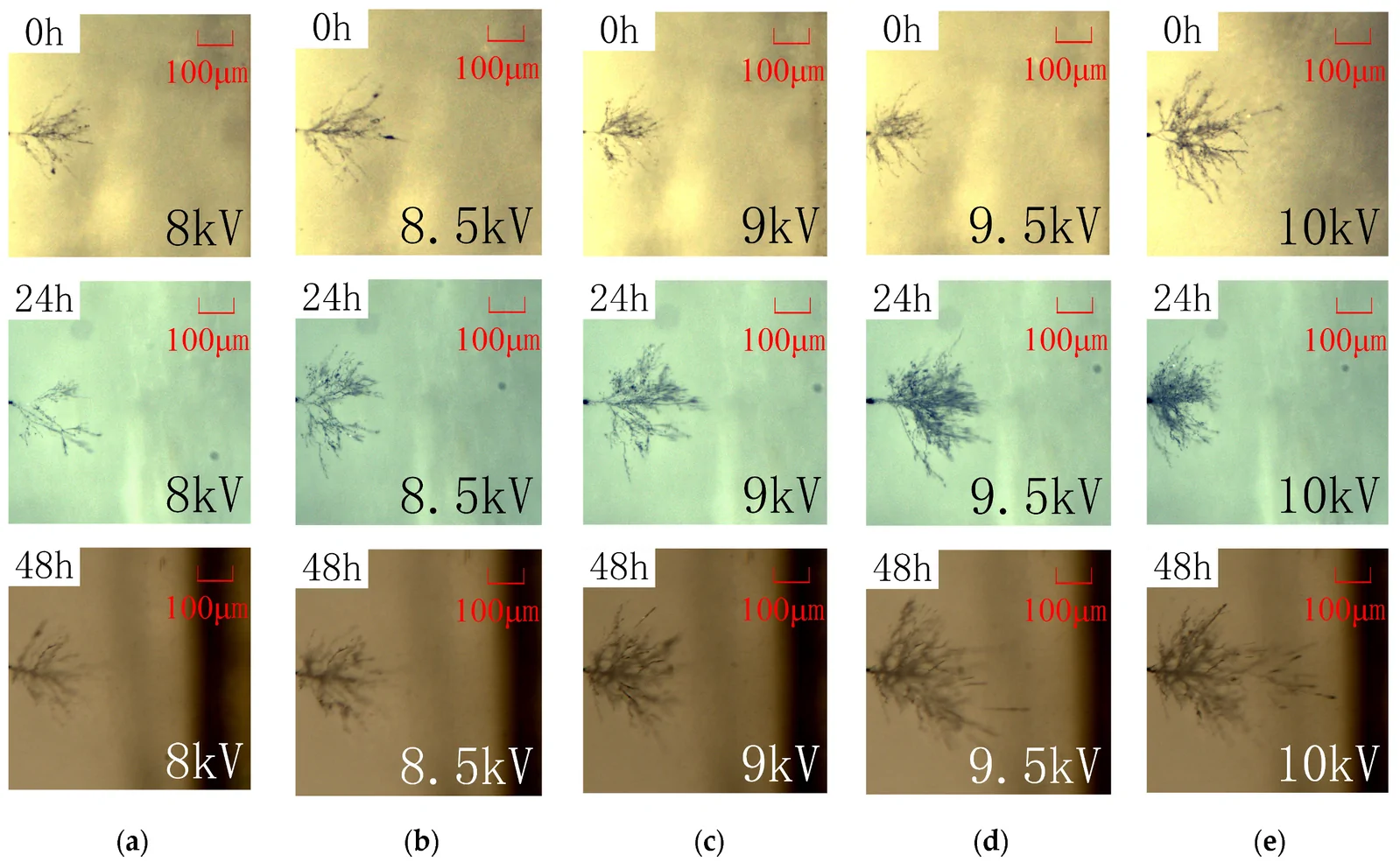
Microscopic changes in electrical trees under different aging times. (**a**) Electrical tree morphology under 8 kV and different aging times; (**b**) electrical tree morphology under 8.5 kV and different aging times; (**c**) electrical tree morphology under 9 kV and different aging times; (**d**) electrical tree morphology under 9.5 kV and different aging times; (**e**) electrical tree morphology under 10 kV and different aging times.

As can be seen from Figure 8, the electrical tree develops obvious branches after 24 h of aging, with a reduced tendency to grow along the electric field direction. At 8 kV, the electrical tree shows a dendritic shape after 24 h of aging and transforms into an arborescent shape when the voltage is further increased, and the microscopic morphology of the electrical tree develops faster after 24 h of aging. After 48 h of aging, the electrical tree morphology appears arborescent and further develops into a pine-like shape. As shown in Table 3, by comparing the length and width of electrical trees, it is found that the growth trend of electrical trees becomes more significant with longer aging time. This indicates that with the extension of aging time, the presence of numerous impurities or defects inside the silicone gel significantly degrades the material’s insulation performance, making the development of arborescent electrical trees more prominent.

**Figure 9:**
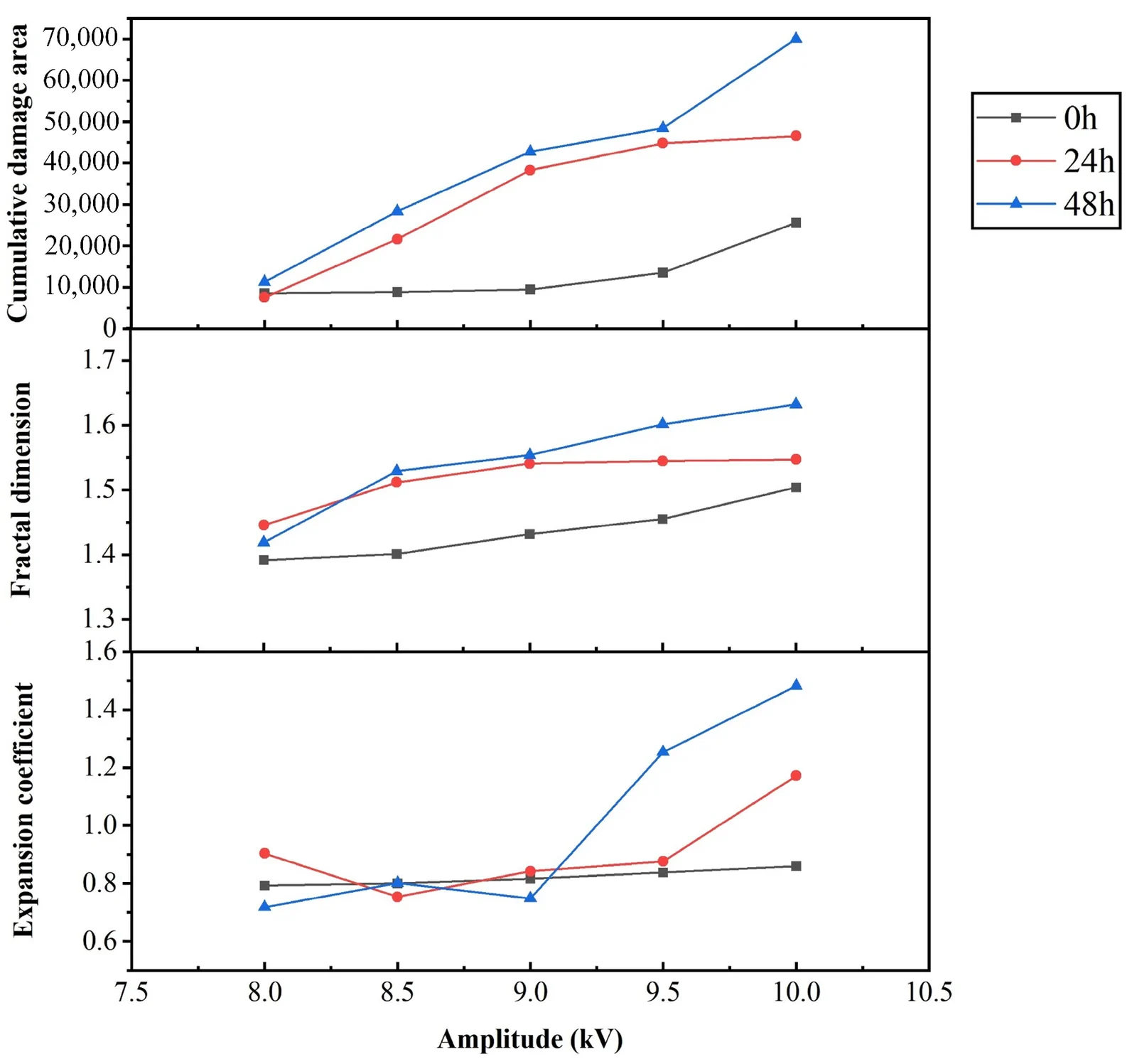
Fractal dimension, expansion coefficient, cumulative damage area of electrical tree under different aging times.

As shown in Figure 9, the expansion coefficient fluctuates slightly with no obvious regularity in the 8 kV–9 kV voltage range. This phenomenon originates from two counteractive influencing factors. On the one hand, impurities and voids inside the material damage the insulating matrix and aggravate partial discharge, which tends to increase the expansion coefficient. On the other hand, thermal aging elevates the molecular crosslinking degree and densifies the internal network structure, inhibiting the longitudinal extension of electrical trees along the electric field and thus decreasing the expansion coefficient. These two factors play opposite roles. The mutual restriction between them ultimately leads to irregular variations in the expansion coefficient.

The cumulative damage area varies similarly to the fractal dimension, both increasing with aging time and voltage. Comparison of cumulative damage areas across aging times shows gentle growth for the unaged specimens, while the 24 h and 48 h aged samples exhibit significant increases in the 8.5 kV–9.5 kV range. This further confirms that aging intensifies material structure damage, thereby accelerating electrical tree development after initiation.

In summary, on the one hand, aging increases the average kinetic energy of gas molecules, promotes gas molecular ionization, and facilitates the initiation of electrical trees [21]. On the other hand, as aging time increases, the silicone gel transforms from a solid–liquid biphasic state to a solid state, and its self-healing capability is greatly weakened, meaning that once an electrical tree initiates, it will propagate more rapidly. Meanwhile, the increased crosslinking degree of molecular chains after aging results in a denser molecular structure, which hinders the development of electrical trees. The interaction of these three effects leads to a complex growth trend of electrical trees.

### 2.2. Characterization and Analysis of Physicochemical Properties of Silicone Gel at Different Aging Stages

Penetration tests were conducted on silicone gel under different aging times. The results are shown in Table 4.

Cone penetration serves as a macroscopic indirect parameter to characterize the crosslinking degree of silicon-based insulating materials. A denser three-dimensional network structure imposes stronger restrictions on the movement of molecular chain segments, which increases the penetration resistance of the cone indenter. For this reason, the cone penetration value is positively correlated with crosslink density. In the cone penetration test, gf represents the real-time penetration resistance borne by the cone indenter during its insertion into silicone gel. A larger gf value indicates higher penetration resistance, greater hardness, and a more compact internal crosslinked network of the material [27]. The peak penetration force of unaged silicone gel at 240 °C reaches 4.7 gf, and the material state corresponding to this value is a typical viscoelastic solid. Its molecular chains are in a moderately crosslinked flexible state and can achieve close micro-level fitting with the device wall through the elastic deformation of molecular chains. After high-temperature aging at 240 °C for 24 h, the penetration sharply rises to 69.2 gf. The reason for this significant change is that high temperature accelerates the cross-linking reaction between molecular chains. The increased crosslinking density restricts the movement of molecular segments, reduces the compliance of the silicone gel, and leads to an increase in hardness. Consequently, the high elasticity of the material is gradually lost, resulting in a sharp decline in packaging fit. When the aging time is extended to 48 h, the penetration increases to 164.4 gf, which is much higher than the initial state. This indicates that under long-term high-temperature conditions, the formed dense crosslinked network further cures, generating tiny pores inside and leading to a continuous decrease in the overall toughness of the material. At this time, the material not only struggles to maintain effective fitting with the device wall but also tends to develop microcracks under the effects of device operational vibration or thermal expansion and contraction. In the experiment, the leakage current of the samples aged for 24 h and 48 h is significantly higher than that of the initial sample, which echoes the content in Section 2.1.4. and reflects the direct correlation between mechanical interfacial matching failure and degradation of electrical insulation performance.

**Figure 10:**
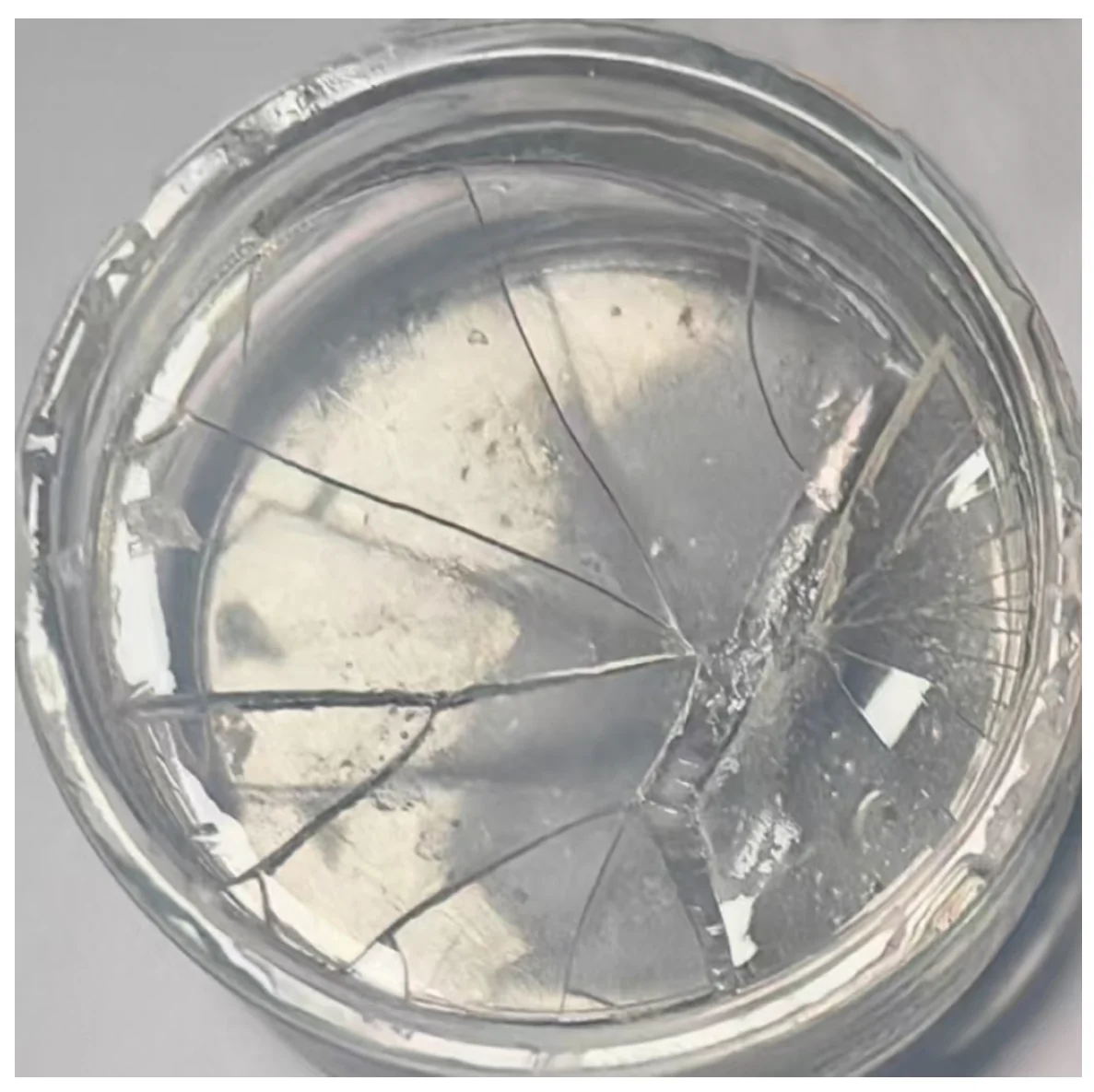
Macroscopic characterization of silicone gel via cone penetration test.

**Figure 11:**
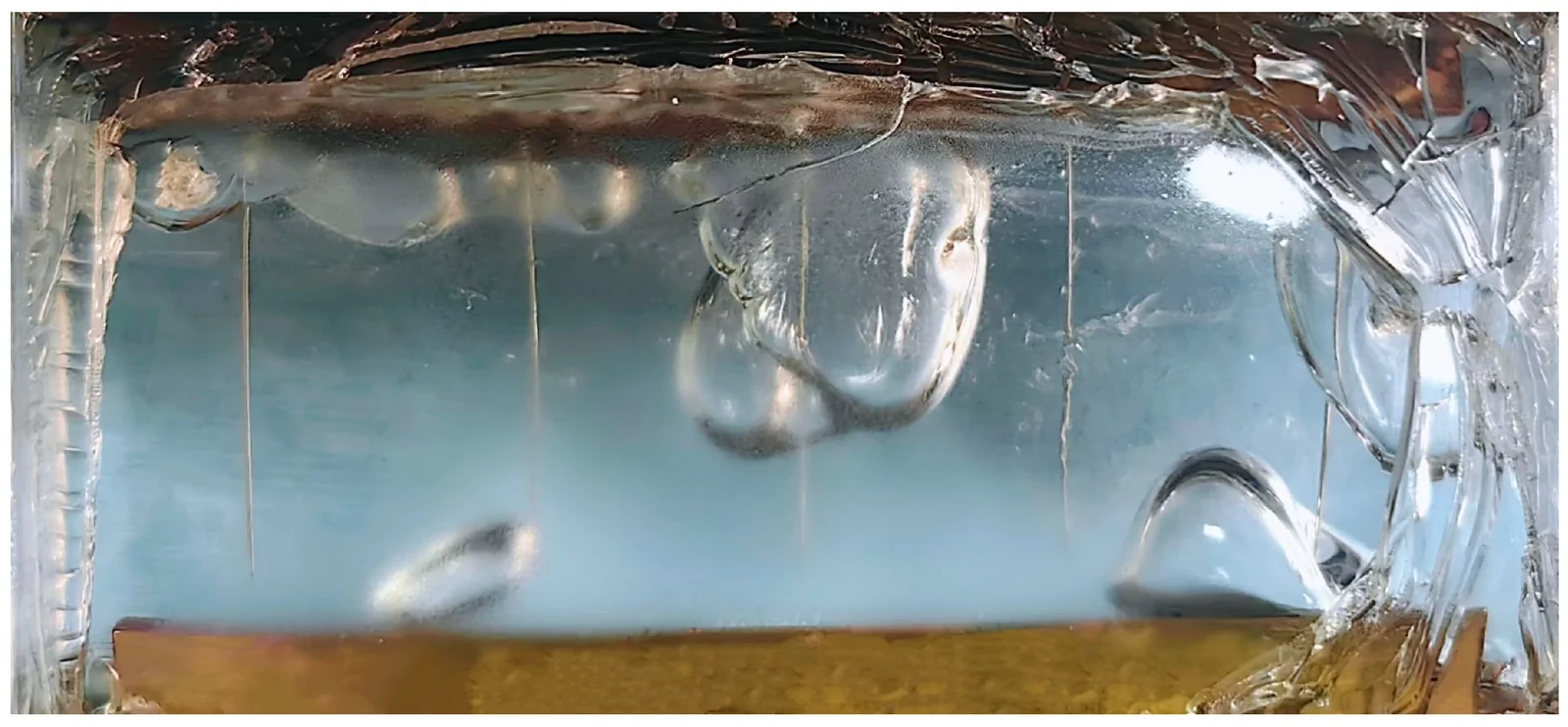
Electrical tree samples after aging at 240 °C for 72 h.

Meanwhile, several macroscopic variations of silicone gel specimens are observed during electrical treeing experiments. As shown in Figure 10, cone penetration tests reveal that a hard shell with a thickness of approximately 2 mm to 4 mm forms on the surface of silicone gel. The inner part of the material remains elastic while the outer surface becomes hardened. After 72 h of aging, thermal expansion and contraction bend the needle tip inside the electrical tree mold inward, and wrinkles appear on part of the sample surfaces. Meanwhile, wrinkles emerge on partial material surfaces, making relevant performance parameters difficult to measure. In addition, visible bubbles can be observed with the naked eye in aged silicone gel at positions relatively far from the needle tip, as shown in Figure 11. In summary, bubble generation and surface hard shell formation inevitably occur in conventional silicone gel samples placed in electrical tree molds during thermal aging. It demonstrates that bubble generation and inhomogeneous structural evolution of the material constitute one of the critical factors leading to the reduction in electrical tree initiation voltage of silicone gel under high-temperature conditions.

In conclusion, the variation law of cone penetration reflects the evolutionary characteristics of the solid–liquid two-phase structure of silicone gel under high-temperature conditions. In pristine unaged samples, the internal liquid-phase components can fill microscopic voids within the material by virtue of their fluidity, so as to improve interfacial fitting and conformability. Nevertheless, high-temperature aging induces volatilization and loss of liquid-phase constituents, which deprives the material of the superior properties originating from the synergy between its solid and liquid phases. Further macroscopic characterizations of silicone gel confirm that bubble generation and inhomogeneous structural variation are among the essential factors responsible for the decline in the electrical tree initiation voltage of silicone gel at elevated temperatures [28].

### 2.3. Discussion on the Electrical-Thermal Synergistic Failure Mechanism of Silicone Gel Under High Voltage and High Temperature

Comprehensive experimental results show that the electrical–thermal failure of silicone gel is not the result of a single factor, but results from the combined effects of high-temperature-induced deterioration of the solid–liquid two-phase structure and subsequent charge dynamic damage initiated by the pulse electric field. It can be seen from the above experiments that the rapid voltage variation at the pulse edge is the core inducing factor triggering electrical failure of silicone gel. That is, the shorter the pulse edge time, the higher the voltage change rate, the severer the electric field distortion, the faster the electron migration speed, and the easier it is to accumulate enough energy to impact the molecular chains. It is found that the intensity of molecular vibration is positively correlated with the pulse amplitude, and the vibration is concentrated in the pulse edge stage, indicating that the electric field strength and change rate jointly determine the energy level of charge movement.

The unique solid–liquid two-phase characteristics of silicone gel are the root cause of its excellent encapsulation performance, but they also make it prone to structural deterioration under high temperature. The experimental results show that under short-term high-temperature aging, the crosslinking degree of molecular chains increases moderately, leading to a denser structure. The breakdown field strength changes slightly, while the initiation voltage of electrical trees is increased instead. However, after long-term aging, the solid–liquid biphasic balance is broken, the volatilization of liquid components leads to the generation of a large number of bubbles, and at the same time, the increase in small molecular impurities and the accumulation of micro-defects cause a sharp drop in breakdown field strength and a sharp deterioration of insulation performance.

At the same time, the harm of high-temperature aging is not only reflected in the decline of electrical performance, but also exacerbates failure through the deterioration of mechanical performance. Cone penetration and leakage current experiments show that after aging, the material changes from a viscoelastic state to a rigid state, and a hard shell is easily formed on the surface, leading to a decrease in the fitting property with the device wall and a significant increase in surface leakage current. The synergistic deterioration of electrical performance and mechanical performance makes the material fail in advance due to problems such as electric field distortion and increased leakage current even if intrinsic breakdown does not occur, which is highly consistent with the phenomenon in practical applications that encapsulation failure precedes material intrinsic breakdown.

The coupling effect of high voltage and high temperature is not a simple superposition of two individual influences. Instead, it forms a mutual accelerating cycle where space charge dynamic damage and structural deterioration reinforce each other. The crosslinking reinforcement effect and defect proliferation effect induced by aging exert opposing impacts on the molecular vibration amplitude. Bubbles and microcavities generated during high-temperature aging aggravate local electric field distortion. Charges injected at the pulse front edge tend to accumulate energy at defect sites more readily, accelerating the propagation of electrical trees and facilitating their initiation. Meanwhile, charged particles under pulsed electric fields continuously bombard molecular chains, triggering chemical bond scission and generating additional defects, which further disrupt the dynamic balance between crosslinking and degradation reactions. In addition, the electric–thermal coupled failure mechanism is also embodied in the loss of the material’s self-healing capacity. Benefiting from its inherent solid–liquid biphasic characteristics, unaged silicone gel can repair tiny local damage via the flow of liquid-phase components, thereby achieving a certain self-repairing effect [29]. After thermal aging, massive volatilization of silicone oil drives the material toward a solid state, resulting in a drastic attenuation of its self-healing capability.

## 3. Conclusions

In this study, 240 °C is selected as the thermal aging temperature, and multiple experimental platforms are established. Combined with characteristic parameters including expansion coefficient, fractal dimension and cumulative damage area, the electrothermal failure mechanism of conventional silicone gel at room temperature as well as high-temperature and high-voltage operating conditions is systematically investigated. This work provides an important theoretical basis and experimental support for the modification and optimization of silicone gel and the reliability improvement of packaging structures for power electronic devices.

(1) The shorter the pulse edge time, the lower the electrical tree inception voltage and the denser the electrical tree propagation, and the larger the fractal dimension and cumulative damage area, which confirms that the space charge dynamic behavior at the edge of the pulsed electric field is the core cause of electrical failure in the material.
(2) Under simultaneous high temperature and high electric field, long-term bombardment by high-energy electrons drastically accelerates silicone gel degradation and premature insulation breakdown. Short-term high-temperature aging leads to a moderate increase in the crosslinking degree of molecular chains, while long-term high-temperature aging disrupts the solid–liquid biphasic equilibrium and accelerates the degradation process of electrical trees.
(3) The electric–thermal coupled failure of silicone gel is not a simple superposition of individual influencing factors. Instead, it arises from the synergistic interaction between space charge damage induced by pulsed electric fields and structural deterioration of the material caused by high temperature. Under high-temperature conditions, the content of free liquid-phase components inside silicone gel declines and the crosslinking degree increases, leading to material structural degradation. Although the breakdown strength is slightly improved in the short term, the material becomes hardened and the leakage current rises. Dynamic charge damage originating from pulsed electric fields aggravates molecular chain scission and the generation of microdefects. The simultaneous deterioration of mechanical and electrical properties ultimately accelerates premature insulation failure of silicone gel.

## 4. Materials and Methods

### 4.1. Electrical Tree Test Platform Under Electrical–Thermal Combined Pulse Electric Field

**Figure 12:**
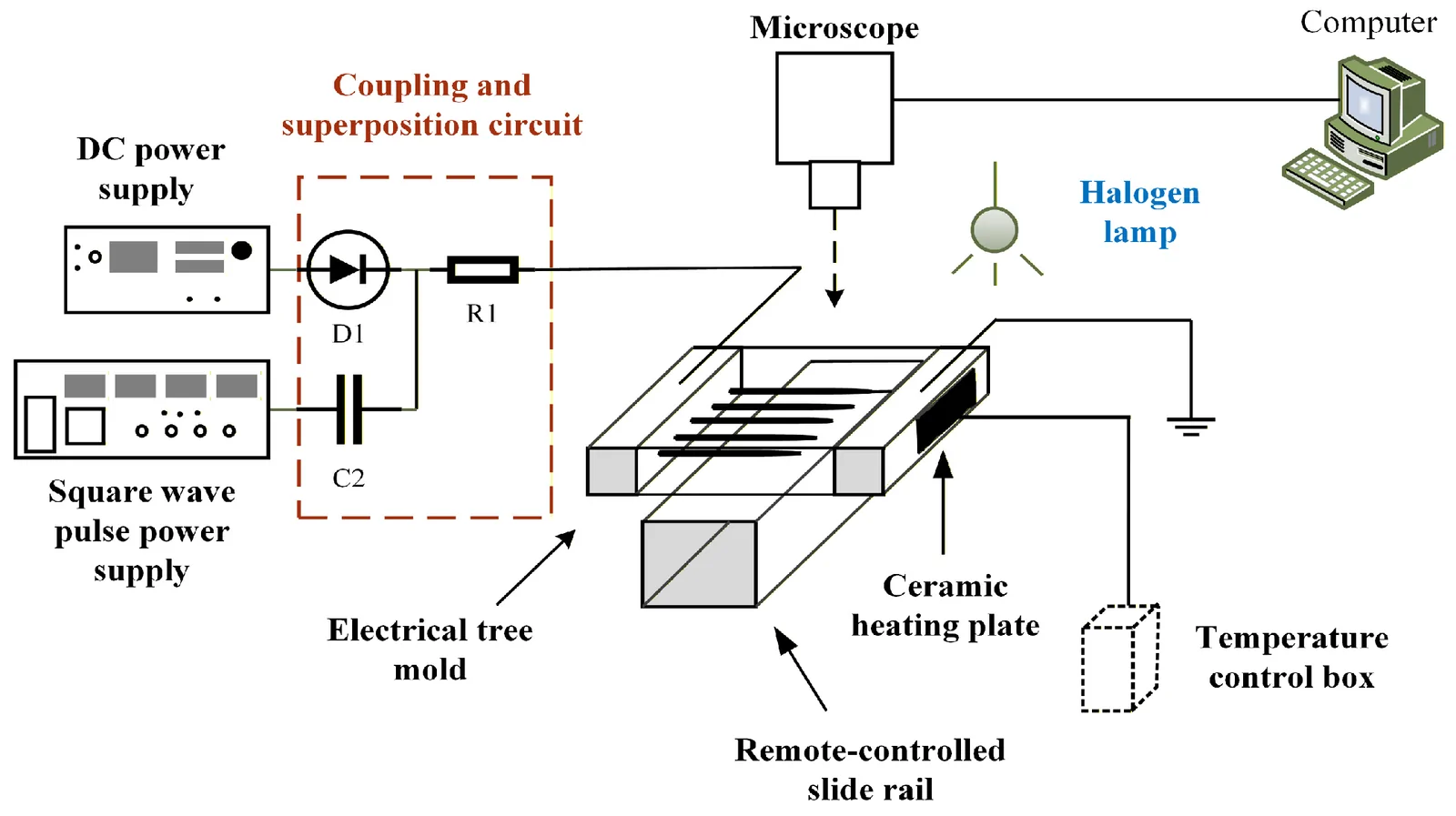
Electrical tree test platform under electro-thermal combined field.

The electrical tree test platform under electro-thermal coupled field, as shown in Figure 12, consists of a high-voltage DC power supply, a square-wave pulse/AC power supply, a coupling and superposition circuit, an electrical tree mold, a remote control slide rail, a halogen lamp, a microscope, a temperature control system, and a computer.

The optical microscope adopted for observation is the Motic SMZ-171TL stereomicroscope (Motic Industries Group Co., Ltd., Xiamen, China), whose magnification can be continuously tuned from 1× to 50×. Dongwen High Voltage Power Supply (Tianjin) Co., Ltd., Tianjin, China manufactures the high-voltage direct current (DC) power supply, which delivers tunable negative DC voltage between 0 and 10 kV. For alternating current (AC) voltage generation, a Trek Model 30/20 A power amplifier (TREK, Inc., Fort Lauderdale, FL, USA) is utilized to magnify the sinusoidal signal from the signal generator by a factor of 3000, yielding a maximum AC output amplitude of 30 kV. The square-wave pulse power supply is the HVP-10B model from Xi’an Lingfengyuan Electronic Technology Co., Ltd., Xi’an, China capable of generating negative square-wave pulse voltages with configurable parameters: amplitude 0–10 kV, frequency 0–10 kHz, pulse edge time 50 ns, and 50% duty cycle.

**Figure 13:**
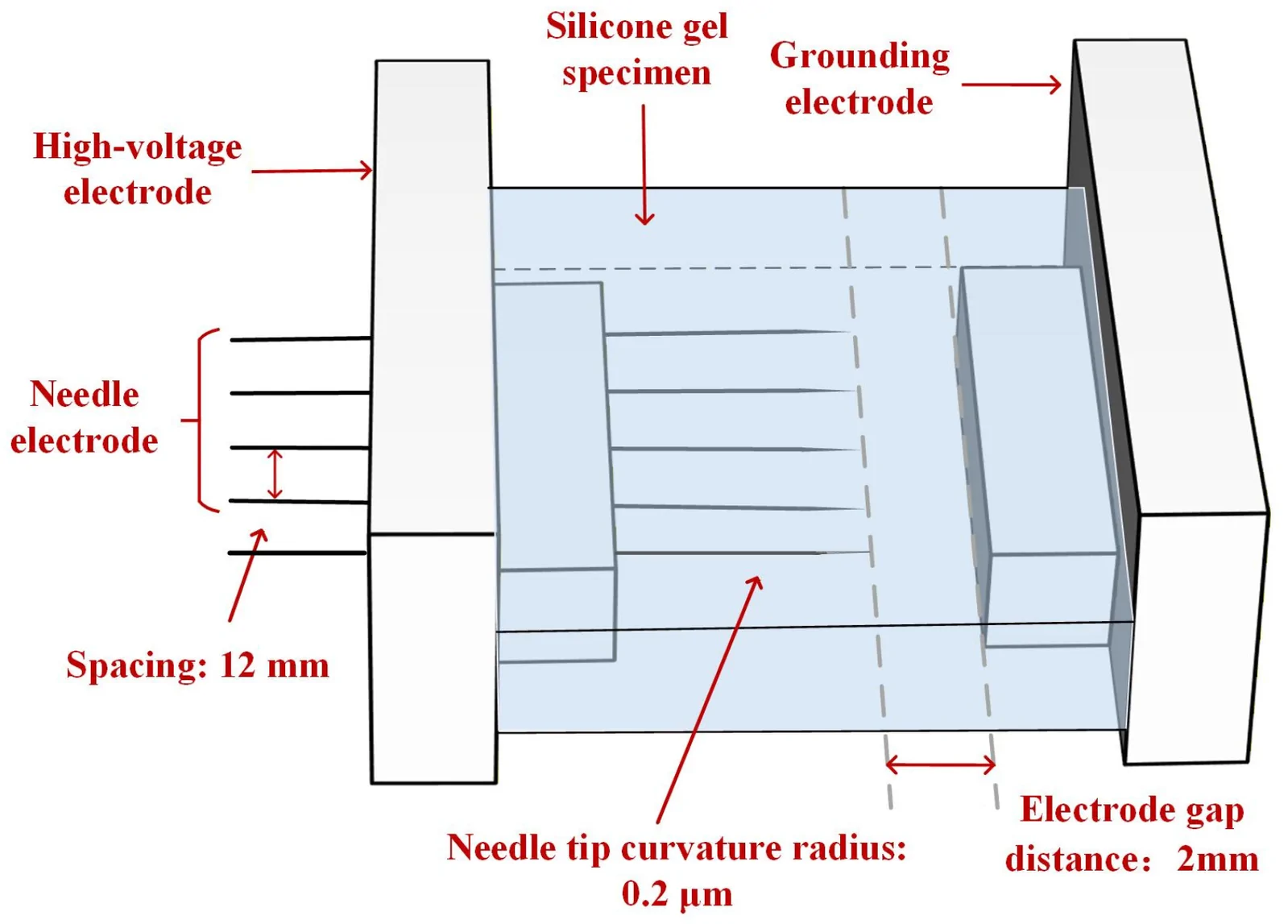
Schematic diagram of the electrical tree mold.

During the experiment, a halogen cold light source is adopted to illuminate the specimen to guarantee sufficient illumination within the field of view. As illustrated in Figure 13, five tiny holes are machined on the high-voltage electrode of the electrical tree mold, where five stainless steel needle electrodes are mounted in sequence. The spacing between adjacent needle electrodes is 12 mm, with a tip curvature radius of 0.2 μm and a tip angle of 30°. The electrical tree mold is fastened onto a remotely controlled sliding rail. Operators can adjust the rail position via a remote controller to bring the five needle electrodes into the microscope’s field of view one after another, enabling observation of electrical tree characteristics at different needle tips. An optical microscope is positioned above the needle tip. Image signals are transmitted to a computer via a charge-coupled device (CCD) camera, facilitating real-time observation and digital storage of electrical tree morphological features originating at the needle tip.

### 4.2. Charge-Excited Molecular Vibration Amplitude Test Platform

The existence of space charge can cause local electric field distortion, affect the electric field distribution inside the insulation, and thereby influence its insulation performance and electrical strength [30]. The electro-acoustic pulse method is a classic method for testing space charge in insulation materials. Its working principle relies on the strong coupling effect between charge carriers and molecular chains, apply a nanosecond pulse electric field to excite charges to drive molecular chains to vibrate, and inferring the charge distribution by testing the acoustic wave intensity generated by the vibration of molecular chains [31].

**Figure 14:**
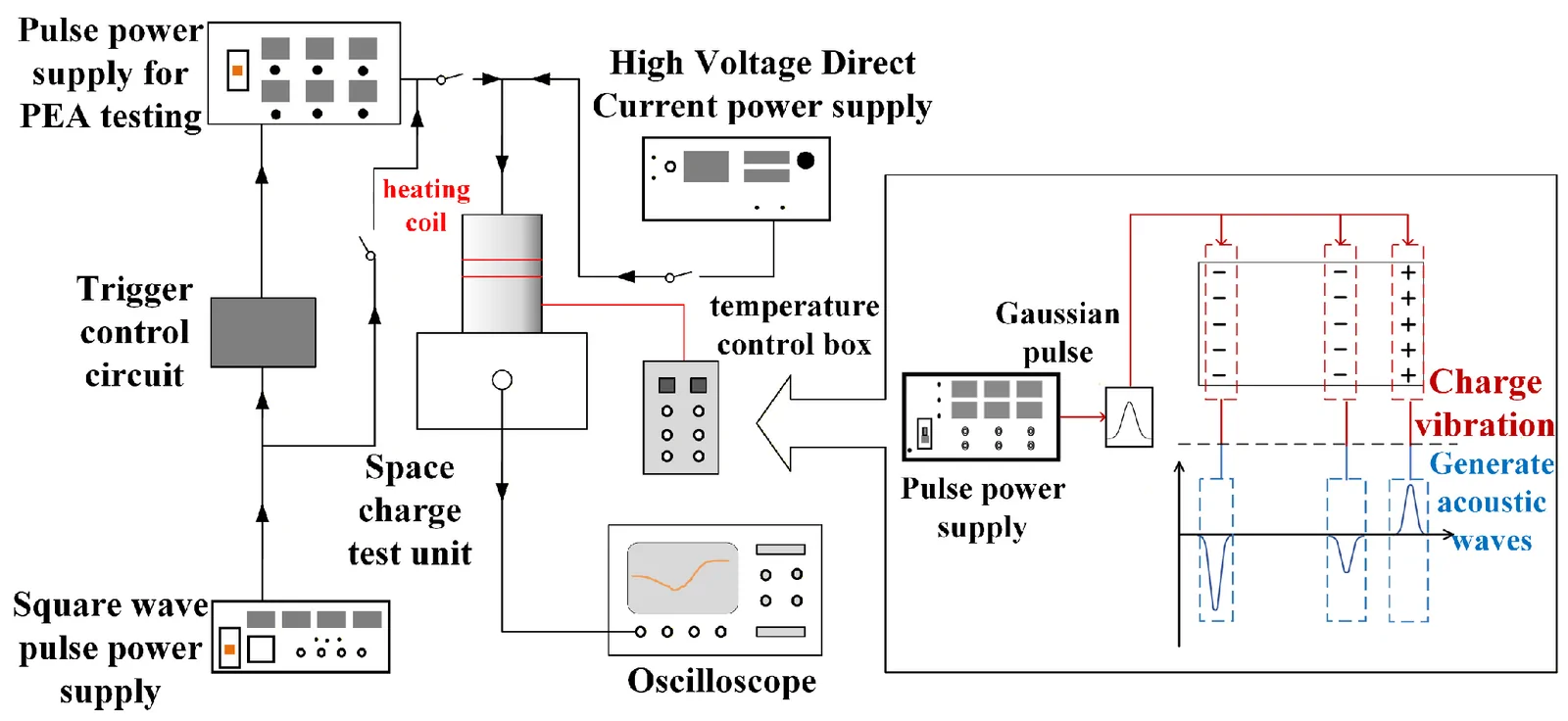
Schematic diagram of the charge-excited molecular vibration and space charge distribution test platform and the electro-acoustic pulse method.

Figure 14 shows the schematic of the charge-excited molecular vibration, space charge distribution test platform, and the pulsed electro-acoustic (PEA) method. Comprising a high-voltage DC power supply, square-wave pulse power supply, PEA test pulse power supply, trigger control circuit, oscilloscope, and acoustic signal test unit, the platform obtains the variation law of electro-acoustic signal amplitude with pulse edge time and amplitude by adjusting pulse edge time and amplitude. It also explores the influence of space charge density on molecular vibration waveforms by applying DC voltage for different durations to alter space charge density.

### 4.3. Electrical Tree Image Processing Methods

Four main methods are used for electrical tree image processing: morphological analysis, fractal dimension analysis, expansion coefficient calculation, and cumulative damage analysis. Electrical tree propagation morphology—a qualitative indicator for assessing the insulating performance of silicone gel and silicone rubber—is categorized into filamentous, dendritic, bush-like, and pine-like morphologies [32].

**Figure 15:**
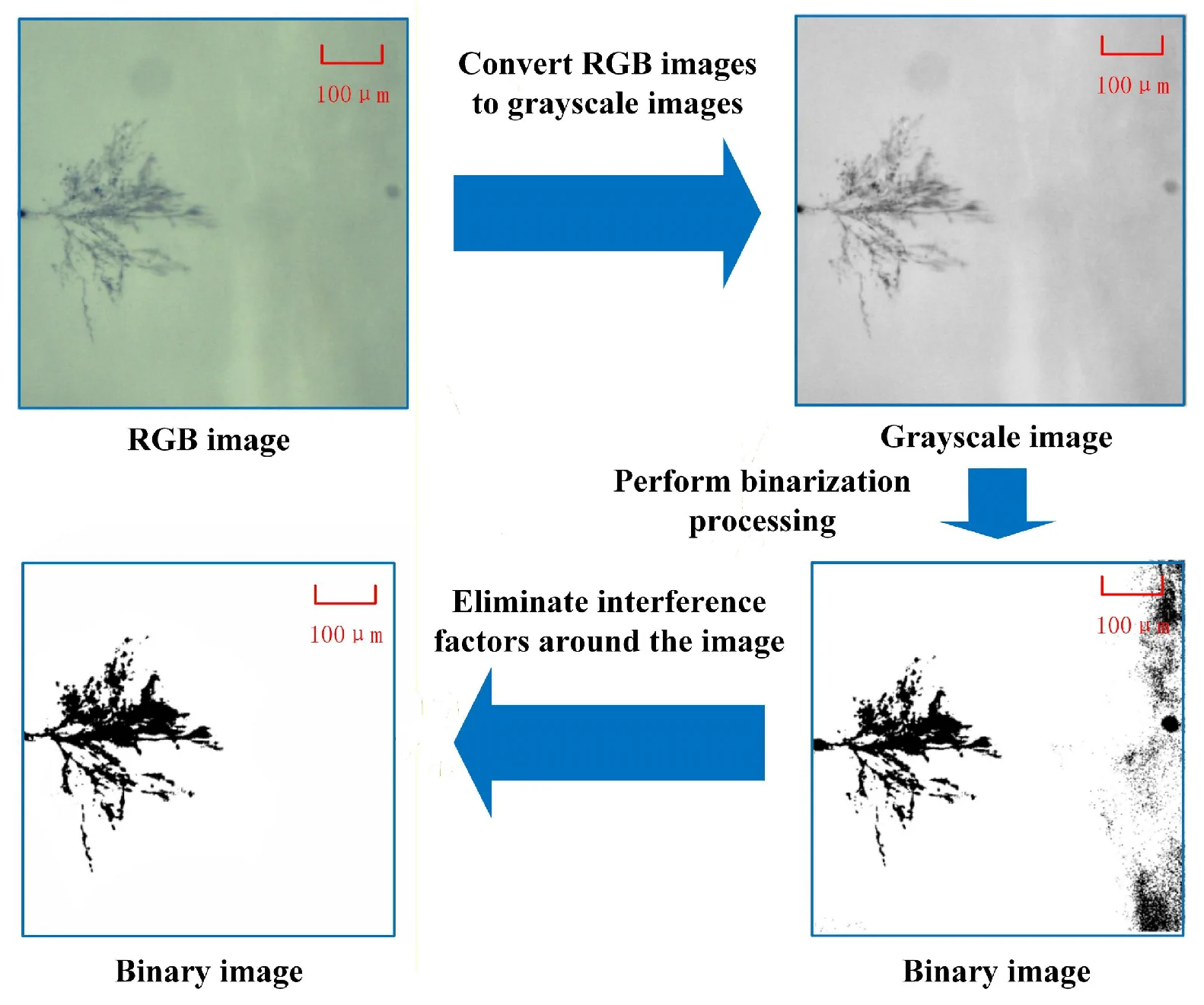
Electrical tree image processing process.

Fractal dimension can quantitatively analyze the shape of electrical trees. To calculate the fractal dimension of electrical trees, it is necessary to process the electrical tree images first, as shown in Figure 15. Firstly, the RGB image needs to be converted into a grayscale image, and then binarization processing is performed to obtain a binary image. Then, the interference factors around the image are excluded, so that the electrical trees are black areas, the background is white areas, the black areas are 0, and the white areas are 1. Then, the binary image is inverted to obtain an inverted binary image. The electrical tree parts are white with a value of 1, and the background parts are black with a value of 0. Then, the image distribution matrix is extracted, and the fractal dimension is obtained by fitting the curve using the above box-counting method.

The length of the electrical tree is the vertical distance from the needle tip to the lowest end of the electrical tree image, i.e., the growth length along the electric field direction. The width refers to the maximum left-right distance of the electrical tree image, i.e., the growth width perpendicular to the electric field direction. The ratio of length to width is the expansion coefficient. A larger expansion coefficient indicates that the electrical tree mainly develops along the electric field direction, while a smaller expansion coefficient indicates that the growth of the electrical tree along the electric field direction is hindered, and it mainly develops perpendicular to the electric field direction [33].

Cumulative damage refers to the area of silicone gel damaged by the electrical tree, reflecting the damage degree. It is obtained by binarizing electrical tree images and calculating the total number of black pixels in the tree channels, serving as a key indicator of silicone material damage caused by electrical trees [34].

## Figures and Tables

**Table 1:** Length and width of electrical trees under different edge times and voltages.

	Length (µm)			Width (µm)		
Edge Time (ns)	9 kV	9.5 kV	10 kV	9 kV	9.5 kV	10 kV
50	304.00	248.00	499.76	364.02	352.02	511.51
100	304.11	292.03	451.50	328.02	296.03	620.05
150	320.00	476.02	463.52	640.00	548.01	536.06
200	448.29	500.02	551.51	363.59	483.50	839.51

**Table 2:** Electrical tree initiation voltage of silicone gel at different aging stages.

	0 h	24 h	48 h
The first needle	6.5 kV	8.5 kV	8.5 kV
The second needle	7.5 kV	7 kV	7 kV
The third needle	6 kV	8.5 kV	7.5 kV
The fourth needle	6 kV	8.5 kV	7.5 kV
The fifth needle	6.5 kV	9 kV	8 kV
average	6.5 kV	8.3 kV	7.7 kV
standard deviation	0.612 kV	0.758 kV	0.570 kV

**Table 3:** Length and width of electrical trees under different aging times and voltages.

	Length (µm)					Width (µm)				
Aging Time (h)	8 kV	8.5 kV	9 kV	9.5 kV	10 kV	8 kV	8.5 kV	9 kV	9.5 kV	10 kV
0	243.77	290.30	304.00	248.00	496.89	305.88	340.23	364.02	352.02	511.51
24	265.63	237.13	321.94	337.51	209.40	221.51	377.75	399.61	440.33	374.62
48	228.13	230.94	281.25	453.14	543.83	309.38	358.77	396.53	543.40	628.15

**Table 4:** Changes in penetration of silicone gel under different aging times.

	0 s	20 s	40 s	45 s	50 s	52.6 s	60 s	70 s	90 s
0 h	0 gf	0 gf	1.5 gf	2.5 gf	4.5 gf	4.7 gf	1.9 gf	0 gf	0 gf
24 h	0 gf	0 gf	0 gf	4.7 gf	44.7 gf	69.2 gf	33.5 gf	0 gf	0 gf
48 h	0 gf	0 gf	0 gf	40.5 gf	139.8 gf	164.6 gf	43.2 gf	0 gf	0 gf

## Data Availability

The data presented in this study are openly available in article.

## Abbreviations

The following abbreviations are used in this manuscript:

DC	Direct Current
AC	Alternating Current
CCD	Charge-Coupled Device
PEA	Pulsed Electro-Acoustic
gf	gram-force
TGA	Thermogravimetric Analysis
